# Knowledge, Attitude, Practice and Barriers Associated with Influenza Vaccination among Health Care Professionals Working at Tertiary Care Hospitals in Lahore, Pakistan: A Multicenter Analytical Cross-Sectional Study

**DOI:** 10.3390/vaccines11010136

**Published:** 2023-01-06

**Authors:** Gulshan Umbreen, Abdul Rehman, Muhammad Avais, Chanda Jabeen, Shakera Sadiq, Rubab Maqsood, Hamad Bin Rashid, Saira Afzal, Richard J. Webby, Mamoona Chaudhry

**Affiliations:** 1 Department of Epidemiology & Public Health, University of Veterinary and Animal Sciences, Lahore 54000, Pakistan; 2 Department of Veterinary Medicine, University of Veterinary and Animal Sciences, Lahore 54000, Pakistan; 3 Department of Veterinary Surgery, University of Veterinary and Animal Sciences, Lahore 54000, Pakistan; 4 Department of Community Medicine, King Edward Medical University, Lahore 54000, Pakistan; 5 World Health Organization Collaborating Center for Studies on the Ecology of Influenza in Animals and Birds, Department of Infectious Diseases, St. Jude Children’s Research Hospital, Memphis, TN 38105, USA

**Keywords:** knowledge, attitude, and practice (KAP), barriers, influenza, influenza vaccination, Health Care Professionals (HCPs), World Health Organization

## Abstract

Health Care Professionals (HCPs), including doctors, nurses, pharmacists, and paramedics, are a high-risk group for influenza infection due to their continuous exposure to patients having a known or unknown history of influenza-like illnesses. Influenza vaccination is the most effective method of primary prevention. This study was conducted to assess knowledge, attitude, practice, and barriers associated with influenza vaccination among HCPs at tertiary care hospitals in Lahore, Pakistan. A multicenter analytical cross-sectional study was conducted among HCPs. Data were collected using a structured questionnaire. All statistical analyses were conducted in R software. A total of 400 HCPs were enrolled, and among these, 67% had a high level of knowledge and 65.5% had a positive attitude towards influenza vaccination. About 51% of HCPs adopted good practices leading to influenza vaccination. Results identified major barriers for influenza vaccinations, including unfamiliarity with vaccine availability (RII = 0.760), insufficient staff for administering the vaccine (RII = 0.649), lack of proper storage (RII = 0.625), safety concerns (RII = 0.613), and cost of vaccine (RII = 0.602). More than half of the HCPs showed a high level of knowledge, a positive attitude, and good practice against influenza vaccination. Despite the positive Knowledge, Attitude, and Practice (KAP) scores and published guidelines, a very low percentage of HCPs were vaccinated against influenza. Many hindering factors were associated with influenza vaccination.

## 1. Introduction

Respiratory tract infections are some of the most common acute illnesses, are classified into two major groups including Upper Respiratory tract (URI) and Lower Respiratory tract Infection (LRI), caused by bacteria, viruses, and mycobacteria [1,2]. Influenza virus remains leading causes of acute respiratory illness in humans and a variety of many other animal species [3,4]. Every year, about 20% of the world’s population gets infected with influenza, resulting in a substantially increased risk of morbidity and mortality [5]. According to the World Health Organization (WHO), an estimated 3 to 5 million cases of severe illness and 290,000 to 650,000 influenza-related deaths occur globally. Influenza is considered one of the most-challenging health problems worldwide [6].

Health care professionals, including doctors, nurses, pharmacists, and paramedics, are a high-risk groups for contracting influenza infection due to their continuous exposure to patients having a known or unknown history of influenza-like illnesses [7]. Health care settings are the ideal environment for rapid spread of influenza [8]. Similarly, the hospital environment could be the source of nosocomial infection of influenza to newly admitted patients [9]. People with Mycobacterium Tuberculosis (MTB) infection are at greater risk of getting influenza due to decreased immunity. This can be further aggravated due to secondary bacterial infection [10]. Patients with MTB influenza co-infection are at an increased risk of death compared to those hospitalized with TB mono-infection [11].

Influenza vaccination is the most-effective method of primary prevention. Thus, Immunization against influenza virus not only reduces the risk of infection among HCPs, but also improves patient safety and reduces morbidity and mortality among the patients [12]. Furthermore, the vaccination of HCPs also protects patients who have not been vaccinated or those who responded poorly to vaccination [13]. During the COVID-19 pandemic, the risk of co-infection of influenza, SAR-CoV-2, and other respiratory viruses has increased. Vaccination is effective to reduce the risk of influenza and its associated socio-economic burden [14]. The World Health Organization (WHO) and Strategic Advisory Group of Experts (SAGE) on immunization have recommended seasonal and pandemic influenza vaccination for HCPs during this pandemic [15]. According to the Centers for Disease Control and Prevention (CDC), vaccines provide 40–60% coverage against influenza by reducing flu illness [16]. Despite these recommendations and the vaccine’s effectiveness, the vaccination rate among HCPs remain globally low [17]. This picture is applicable globally, ranging from >90% (several Central American countries) to <5% (Southeast Asia) [18].

In Pakistan, a lack of proper storage, a lack of sufficient staff to administer the vaccine, side effects, safety concerns, cost of the vaccine, doubts about the effectiveness of the vaccine, and the fear of needles are factors/barriers preventing HCPs from getting vaccinated [7]. In Pakistan, there are currently no specific/published guidelines for influenza vaccination in health care settings [19]. In Pakistan, few studies have been conducted on influenza. Most of these were conducted to estimate the influenza A virus burden and risk factors in the general population [20,21]. Data on knowledge, attitude, practice, and barriers regarding influenza vaccination among HCPs in Pakistan are very limited [12,19]. This KAP survey of influenza vaccination among HCPs was the first study conducted in Lahore, Punjab, Pakistan. Hence, the current study was planned to reduce the gap of data, and a KAP survey was designed to collect data about the knowledge, attitude, practice, and barriers regarding influenza vaccination among HCPs. The current data generated could be used to devise strategies to improve vaccination coverage among HCPs and reduce influenza-related morbidity and mortality.

## 2. Materials and Methods

### 2.1. Study Design and Population

**Figure 1:**
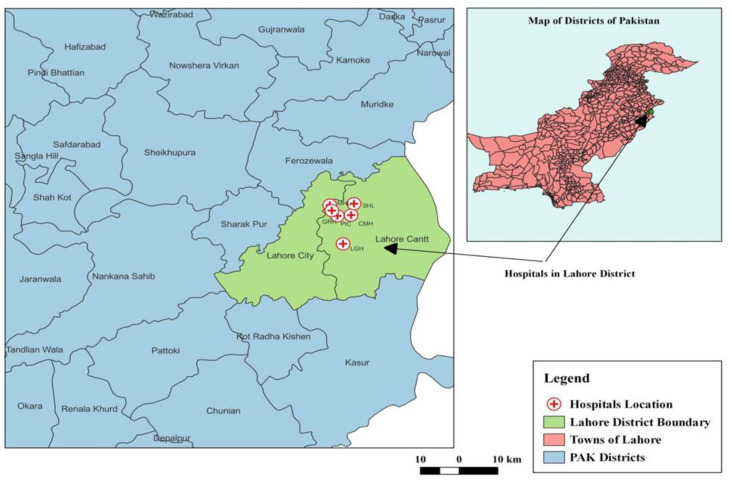
Study map area of the hospitals’ location in Lahore District.

A multicenter analytical cross-sectional study was conducted to assess knowledge, attitude, practice, and barriers regarding influenza vaccination among HCPs working in 6 major tertiary care hospitals (Mayo Hospital Lahore (MHL), Lahore General Hospital (LGH), Punjab Institute of Cardiology (PIC), Shalamar Hospital Lahore (SHL), Combined Military Hospital (CMH), Lady Aitchison Hospital (LAH)) in Lahore District, Pakistan. Lahore is the capital city of Punjab Province located at 31°32′59″ latitude and 74°20′37″ longitude [22] and the 2nd-most-populous city of Pakistan. It is situated in the northeastern part of the country. The geographical locations of the hospital addresses were located on Google maps. A dot map was produced using the QGIS software Version 3.2 (Open Source Geospatial Foundation Project, Boston, MA, USA) (Figure 1). All these hospitals were selected due to the availability of access to the facilities. There is no influenza vaccination program currently going on in Lahore, Pakistan, and currently, there is no mandatory vaccination for influenza included in any vaccination program, especially for HCPs. Access was made available to collect the data. All respondents were very co-operative. The international guidelines by the World Health Organization (WHO) and Centers for Disease Control and Prevention (CDC) are available online. 

The healthcare professionals enrolled in the current study were between the ages of 18 and 50 years, and they perform duties in pulmonology or microbiology in medical departments or shared sections that have direct contact with patients (e.g., medicine, Outpatient Department (OPD), X-ray rooms, and pharmacies). Those healthcare professionals suffering from any chronic co-morbidity were excluded.

### 2.2. Survey Instrument and Data Collection Procedure

The study was conducted according to guidelines of the Declaration of Helsinki and approved by the Institutional Review Committee for Biomedical Research, University of Veterinary and Animal Sciences, Lahore, Pakistan (Letter No. 127/IRC/BMR). Written informed consent was obtained from all study participants, who were aged 18 years or older. All participants were given a consent form in English and verbally briefed about research objectives and data collection procedures. The questionnaire was adopted from a previously validated (Cronbach’s alpha = 0.87), self–administered questionnaire [12], modified according to the objectives of the current study. The questionnaire comprised closed-ended or multiple-choice questions about knowledge, attitude, practice, and barriers regarding influenza vaccination. Data were collected during a face-to-face interviews by a trained research team (Registration No: A-67641) registered with Pakistan Nursing Council. Each interview lasted approximately 20 min. Questions were written in English, which were easily understood by the participants. A unique coded identification number was given for each questionnaire to keep data confidential. A total of 400 HCPs were enrolled in current study. The study protocol followed the Strengthening the Reporting of Observational Studies in Epidemiology (STROBE) statement.

The questionnaire comprised five sections: the first section of the questionnaire had demographic data with 13 items; the second section included questions about the participant’s knowledge with 15 items; the third section consisted of questions related to the attitude of respondents (10 items); the fourth section had questions about practice (8 items); the last section included questions regarding barriers regarding influenza vaccination (11 items). A pilot study was conducted in 30 HCPs to assess the validity of the questionnaire tool. Reliability and internal consistency were assessed using Cronbach’s alpha test. The overall reliability of the tool (Cronbach’s alpha) was 0.7.

### 2.3. Statistical Analysis

The datasets were entered into the EpiData software (Version 3.1, Odense, Denmark, available at http://www.epidata.dk/) and validated for errors and inconsistencies by randomly checking the digital data with the hard copy record, then exported to Microsoft Excel (Version 2016, Microsoft Office, USA) for further processing. All statistical analyses were conducted in the R software (Version 4.2.1, R Foundation for Statistical Computing, Vienna, Austria). Categorical variables were measured as frequencies and proportions.

#### 2.3.1. KAP Scores

A three-point Likert scale (3 = yes, 2 = no, 1= don’t know) was used to assess knowledge about thirteen items. A five-point Likert scale (5 = strongly agree, 4 = disagree, 3 = neutral, 2 = disagree, 1 = strongly disagree) was used to assess attitude about ten items and practice about eight items (5 = very frequently, 4 = frequently, 3 = occasionally, 2 = rarely, 1 = never).

Scores were summed to generate an individual total knowledge score (range 1–45), total attitude score (1–50), and total practice score (1–40) for each respondent. These summed scores were divided by the number of respondents (N = 400) to calculate the mean score for each section (knowledge, attitude, and practice) [23].

#### 2.3.2. Criteria for the Categorization of the KAP Score

The scores of knowledge, attitude, and practice obtained were categorized. Overall knowledge score was categorized as high level of knowledge ≥ 75% and low level of knowledge < 75%. The attitude of respondents was categorized as positive: total score > mean (37.3) and negative: total score < mean (37.3). The practice of respondents was categorized as good: total score > median (40) and poor: total score < median (40) [24].

#### 2.3.3. Composite of Total KAP Score

The scores of knowledge, attitude, and practice obtained were categorized as high, low, positive, negative, good, and poor, respectively. The scores of each respondent were assessed by considering their composite scores on the Knowledge, Attitude, and Practice (KAP) scale. A KAP response with high, positive, and good score was given a value = 1, while low, negative, and poor scores were given a value of 2. Similarly, the trends of the KAP scores were aggregated into 8 possible groups as follows: high knowledge, positive attitude, and good practice (1,1,1) KAP scores represented by 1, low knowledge, negative attitude, and poor practice (2,2,2) KAP scores represented by 2, high knowledge, negative attitude, and good practice (1,2,1) KAP scores represented by 3, low knowledge, negative attitude, and good practice (2,2,1) KAP scores represented by 4, low knowledge, positive attitude, and good practice (2,1,1) KAP scores represented by 5, high knowledge, positive attitude, and poor practice (1,1,2) KAP scores represented by 6, low knowledge, positive attitude, and poor practice (2,1,2) KAP scores represented by 7, and high knowledge, negative attitude, and poor practice (1,2,2) KAP scores represented by 8, respectively. In addition, the frequency (percentages) of respondents that were represented by 1, 2, 3, 4, 5, 6, 7, and 8 were aggregated. All those having good ratings for knowledge, attitude, and practice were rated as good KAP scores, and those that had a poor score on the three scales were rated as poor KAP scores [25].

#### 2.3.4. Barriers Regarding Influenza Vaccination

A five-point Likert scale was used to assess barriers regarding influenza vaccination.

N represents the total number of respondents; 5 = the highest weighted score (1, 2, 3, 4, 5); n1 = the number of participants who selected “strongly disagree”; n2 = the number of participants who selected “disagree”; n3 = the number of participants who selected “neutral”; n4 = the number of participants selecting “agree”; n5 = the number of participants who selected “strongly agree.” Additionally, to prioritize barriers among health care professionals, a Relative Importance Index (RII) was also calculated. RII = (1n1 + 2n2 + 3n3 + 4n4 + 5n5)/5N (0 ≤ RII ≤ 1). The score for each factor was calculated by summing up the scores given to it by the participants. The value of the RII ranged from 0 to 1. The value closest to 1 was ranked as the main barrier to influenza vaccination as compared to the others.

#### 2.3.5. Inferential Statistics

A Shapiro–Wilk normality test was applied to determine the nature of the data distribution of the KAP score. Spearman’s rank correlation coefficient was used to analyze relationship among the knowledge, attitude, and practice scores. A Whitney U-test (Wilcox rank-sum test) was used to determine the statistically significant difference between the two independent groups, Kruskal–Wallis test was used to determine statistically significant differences in three or more independent groups. A *p*-value < 0.05 was considered significant, and a *p*-value < 0.01 was considered highly significant.

A Fischer exact test was applied to identify the significant factors hindering influenza vaccination. A *p*-value < 0.05 was considered significant, and * marks highly significant results. The Fisher exact test was conducted between influenza vaccination in the last 6–12 months (Yes, No) and for barriers to influenza vaccination (SDA, DA, N, A, SA).

## 3. Results

### 3.1. Socio-Demographic Characteristics of Respondents

The socio-demographic characteristics of the respondents are described in detail in Table 1. The study revealed that over half of the respondents were female nurses (54.1%), followed by physicians (45.2%). The majority of the respondents (283 (70.75%)) were females, and 117 (29.25%) were males. Most respondents (80%) were young adult females, while the fewest respondents came from the age ranges of 31–35 years and over 35 years. In addition, only 23 (19.7%) male HCPs and 60 (21.2%) female HCPs had a history of influenza vaccination in the past 6–12 months.

### 3.2. Health Care Professionals’ Knowledge about Influenza Vaccination

Regarding knowledge of HCPs about influenza vaccination, 85.5% HCPs had responded that the “influenza vaccine is effective in preventing the ‘flu”, while 75.8% also responded that the World Health Organization (WHO) gives the recommendation on influenza vaccination of health professionals. Moreover, a majority (43%) of the respondents were aware of the guidelines published by the WHO Advisory Committee on Immunization Practices (ACIP) for influenza immunization. About 60.5% of participants had a perception that vaccination did not provide 100% protection against the flu. A total of 83% HCPs knew that they can transmit influenza to their patients (Table 2).

Most of the respondents (67%) had a high level of knowledge (≥75%), and 33% respondents had a low level of knowledge, having scores of <75%, (Table 3).

### 3.3. Health Care Professionals’ Attitude towards Influenza Vaccination

Among all enrolled participants, 63.7% agreed that vaccines are effective at preventing influenza. Over half of the respondents (52%) believed that vaccination lowers the risk of hospitalization and death. More than half of respondents (60.7%) thought that the vaccine may decrease the days of illness with influenza. The response to items in the questionnaire showed that the majority of the respondents agreed with the statements that vaccinating healthcare personnel protects patients (54.7%) and those healthcare personnel (40.2%) should get vaccinated against influenza every year. Approximately 51.5% of respondents believed that the influenza vaccine should be part of routine medical practice (Table 4). The scores related to the attitude of respondents are summarized (Table 3). It was found that more than half (65.5%) of the respondents had a positive attitude (total score > mean) towards influenza vaccination, and 34.5% of respondents had a negative attitude (total Score < mean) towards influenza vaccination

### 3.4. Health Care Professionals’ Practice Regarding Influenza Vaccination

Most respondents (51%) had a good practice regarding influenza vaccination, where 49% of respondents had poor practice regarding influenza vaccination (Table 3).

The respondents practice with respect to influenza vaccination was determined in this study (Table 5), and it was found that 35.5% the HCPs rarely go to a health facility when they have signs of a cough, cold, and/or sore throat. Moreover, most respondents (36.2%) wear masks when having signs of a cough and/or cold. However, only 23.2% of the respondents received the influenza vaccine frequently, while 44.2% of the HCPs never received the influenza vaccine on regular basis.

### 3.5. Composite Total Knowledge, Attitude, and Practice Score

**Figure 2:**
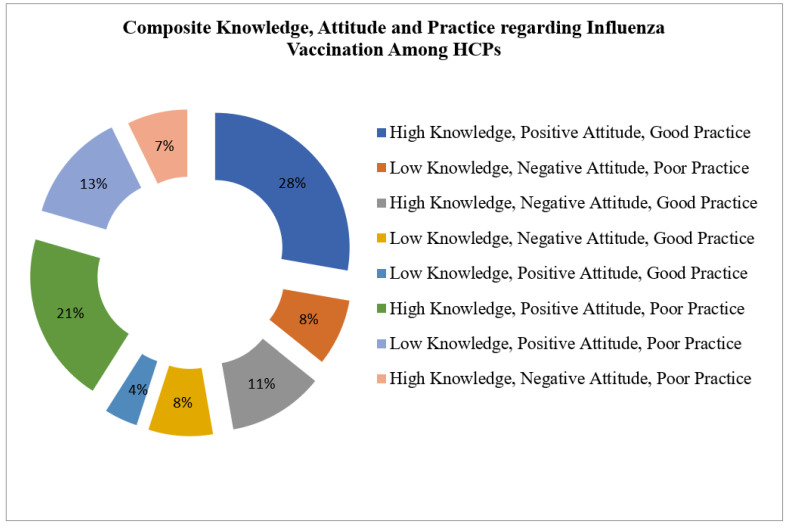
Composite total KAP score.

Respondents were further classified based on their KAP scores using the rating system previously mentioned. The composite KAP of the respondents revealed that (28%) had a positive KAP score, while 21% showed high knowledge, positive attitude, and poor practice. On the other hand, 13% showed low knowledge, positive attitude, and poor practice, while 11% showed high knowledge, negative attitude, and good practice. In addition, 8% showed low knowledge, negative attitude, and poor practice, while the other 8% showed low knowledge, negative attitude, and poor practice. The study also showed that 7% showed high knowledge, negative attitude, and poor practice. Only 4% of the respondents showed low knowledge, positive attitude, and good practice (Figure 2).

### 3.6. Correlation among Knowledge, Attitude, and Practice

Although the positive association was weak, a statistically significant correlation was found between knowledge-attitude and knowledge-practice, there was a weak negative and not statistically significant correlation between the knowledge and practice variables. A *p*-value < 0.05 was considered significant, and a *p*-value < 0.01considered highly significant (Table 6).

### 3.7. Health Care Professionals’ Knowledge, Attitude, and Practice Score Regarding Influenza Vaccination against Gender, Age, Education, Profession, and Job Experience

The current study revealed a highly significant difference in knowledge (*p* = 0.000), attitude (*p* = 0.000), and practice (*p* = 0.000) according to gender. it indicated that females had a high level of knowledge, positive attitude, and good practice as compared to males. There were also highly significant differences in knowledge (*p* = 0.000) and practice (*p* = 0.008) as per age, but there were no statistical differences in attitude (*p* = 0.94). These results showed that respondents 21–25 years old have high levels of knowledge, positive attitude, and good practice compared to other age groups. On the other hand, the respondents’ attitude (*p* = 0.638) did not show a statistically significant difference among professions. The study further showed that respondents having 1–2 years of working experience had a high level of knowledge, However working experience was not statistically different attitude (*p* = 0.915) and practice (*p* = 0.241) of respondents (Table A1) (see Appendix A).

### 3.8. Barriers to Influenza Vaccination

When exploring the HCPs’ justification/barriers for not being vaccinated against influenza, about 30.5% agreed that there is a lack of proper storage area for vaccination. However, the majority (37.3%) disagreed with the statement that influenza vaccine is not compulsory for HCPs. About 55.5% stated that not everyone was familiar with the availability of influenza vaccination at their institution. Furthermore, 9 barriers out of 11 were highly significant by the Fisher exact test (*p* = 0.000). In addition, when calculating Relative Importance Index (RII), barriers were categorized from most important to least important. When finding a score of RII = 0.760, not everyone was familiar with the availability of vaccines at their institute, and this was categorized as the number-one barrier. Furthermore, insufficient staff to administer vaccines (RII = 0.649) was ranked as the number two barrier. Further details are given in (Table A2) (see Appendix A).

## 4. Discussion

Influenza is a highly contagious disease, and HCPs are at a higher risk of becoming infected and also serve as carriers, transmitting influenza to their patients. Influenza vaccination is the most-effective method for the prevention of influenza virus infection and its associated complications. This study was conducted to assess the knowledge, attitude, practice, and barriers regarding influenza vaccination among HCPs. Knowledge, attitude, and practice are aspects that represent behavioral domain. It is well understood that knowledge and attitude can have an impact on an individual’s prevention practices [26]. Published studies show that vaccinating HCPs against influenza is an effective intervention for preventing infections, reducing transmission to patients, and lowering mortality and morbidity among vulnerable groups. Vaccination also reduces absenteeism and improves HCPs’ health [12]. Despite ACIP and CDC recommendations and HCPs being at higher risk of infection, this study found low rates of influenza vaccination (20.75%) among health care professionals in Lahore, Pakistan. These findings are consistent with earlier published research findings of lower rate of influenza vaccination among HCPs. Furthermore, the current study’s influenza vaccination rate among HCPs is likely the lowest when compared to the available literature from different regions of the world, namely Kuwait (67.2%), Oman (46.6%), The Kingdom of Saudi Arabia(KSA) (38.0%), France (30.6%), and The United Arab Emirates (UAE) (24.7%) [27]. The low rate of influenza vaccination among HCPs in Lahore, Pakistan, is likely related to the unfamiliarity of vaccine availability, cost of the vaccine, and some concerns/beliefs about influenza vaccination. Various socio-demographic factors, e.g., age, gender, marital status, and education level, have been found to be associated with vaccine hesitancy during the pandemics of HINIpdm09 and COVID-19 [28,29,30]. In our study, vaccine hesitancy was frequent in young people (age 21–30) compared to older age (>30 years). As per WHO, the rational model of health promotion assumes that high knowledge would translate to a positive attitude and, as a result, lead to good behavior; however, in reality, the transition is not straightforward and is dependent on a number of factors [31]. In this regard, the study compared distributions of respondents by composite knowledge, attitude, and practice performance. In our study, 28% had a positive rating on the KAP score, while 7% had a negative rating on the KAP score. However, more than half who were in various categories required interventions to improve knowledge, attitude, and practice regarding influenza vaccination. In our study, the majority of the respondents (48.7%) knew that vaccination should be administered every year and were also aware of the WHO guidelines’ recommendation for influenza vaccination. Similarly, in earlier published studies, HCPs understood that they were included in the high-risk group and should be vaccinated annually. They knew WHO guidelines for influenza vaccination. Further HCPs also understood that their role in disease transmission and that vaccination helps to stop the spread of infection to others [32,33]. The summation of this attitude revealed that most HCPs (65.5%) had a positive attitude toward influenza vaccination. This could be due to the high level of knowledge of health care professionals. These results correspond to a study in which 67% had a positive attitude towards influenza vaccination [34]. These results are also consistent with a study conducted by Mojamamy et al. [35]. Health care professionals are exposed to numerous infections and diseases given their nature of work. Hence, the current study revealed some preventive strategies used by HCPs. Most respondents (36.2%) frequently wore mask, and 46.2% washed their hands very frequently. Hand washing is a key step to prevent cross-infections at health care facilities. Summation of practice scores revealed that most HCPs (51%) had good practice regarding influenza vaccination. The result of the current study indicated a high level of knowledge and a positive attitude, which ultimately lead to good practice. These positive KAP scores are important for the prevention and control of influenza and other associated complications. These results are also consistent with a study conducted by Mojamamy et al. [35], which showed that 58% of the respondents had a positive practice. The results of the current study revealed the highly significant difference in knowledge (*p* = 0.000), attitude (*p* = 0.000), and practice (*p* = 0.000) according to gender and indicated that females had high level of knowledge, a positive attitude, and a good practice as compared to males. Females showed more concern and response towards influenza as compared to males, which shows a great difference between them. These results contradict a previous study in which male doctors possessed better knowledge (8.05 ± 1.39, *p* = 0.003) than female doctors [12]. Further, the study showed respondents having 1–2 years of work experience and high levels of knowledge (*p* ≤ 0.001), work experience was not statistically different according to the attitude (*p* = 0.915) and practice (*p* = 0.241) of the respondents. These results are similar to a study wherein new graduates, with 1–2 years of job experience, had significantly (*p* = <0.001*) better knowledge compared to others [19]. In our study, the top-ranked barrier to influenza vaccination among HCPs was that not everyone was familiar with the availability of the vaccine at their institute (RII = 0.760). Similarly, a study conducted by Khan et al. in Pakistan [19] reported the top-ranked barrier to vaccination (RIWF = 0.71) among HCPs to be a lack of awareness of the availability at their institute. These results are similar to a study conducted by James et al. in Sierra Leone [36]. This may be due to the lack of orientation programs and public health activities at the hospitals, which may reduce the health care professionals’ interest in investigating the availability of such services at their institutes. In this study, the other barriers found to influenza vaccination were a lack of proper storage, a lack of sufficient staff to administer the vaccine, side effects and safety concerns, the cost of the vaccine, doubts about the effectiveness of the vaccine, and the fear of needles. Similarly, earlier previous studies have also reported these barriers among health care professionals [12,19,37,38]. In Pakistan, the majority of HCPs are Muslim, and they have some concerns and beliefs about whether vaccines are “halal” or not. Regarding the affordability of vaccines, one of the barriers is the cost of the vaccine (influvac tetra), which is about USD 8.8431 in 1989 PKRs. Due to the high cost, most of the HCPs were not concerned with this. Cost effectiveness is crucial for the acceptance of a vaccine. The Health Ministry of Punjab in Pakistan has ensured the availability of the influenza vaccine by reducing the cost of the influenza vaccine at their institute, which encourages health professionals to get vaccinated [37].

The findings of the current study could help devise an immunization policy for HCPs in Pakistan by the Health Ministry. The Health Ministry of Punjab, Pakistan, should ensure the availability of influenza vaccines at each health care facility free of cost to HCPs to avoid hesitancy related to the high cost of the influenza vaccine. This will help the HCPs maintain their health and stop the spread of such diseases to their patients. Furthermore, educational seminars and awareness campaigns should be arranged for HCPs.

The limitation of our study is that the results cannot be generalized to all HCPs in Pakistan, as this study only included HCPs from Lahore, Punjab. Another limitation is, at the time of the design, it was not comprehended by the investigator that the option of “don’t know” could affect the validity of the study. We recommend excluding the “don’t know” option from the response for the section about the knowledge of influenza vaccination, as it might affect the validity of study.

## 5. Conclusions

In the current study, more than half of HCPs showed a high level of knowledge, a positive attitude, and a good practice regarding influenza vaccination. Despite the positive KAP scores, and published guidelines and recommendations, a very low percentage of HCPs in our hospitals were vaccinated against influenza. Unfamiliarity with vaccine availability, insufficient staff to administer the vaccine, side effects and safety concerns, cost of the vaccine, doubts on the effectiveness of the vaccine, and the fear of needles were some of the main barriers to influenza vaccination. These barriers should be ruled out by using various strategies such as arranging awareness, educational seminars, and sessions about vaccinations.

## Figures and Tables

**Table 1:** Socio-demographic characteristics of respondents according to gender (N = 400).

Demographics		Male (117) (29.25%)	Female (283) (70.75%)	Total
Age	21–25 Years	76 (64.9%)	174 (61.5%)	250 (62.5%)
	26–30 Years	32 (27.3%)	73 (25.8%)	104 (26%)
	31–35 Years	5 (4.27%)	25 (8.83%)	30 (7.5%)
	>35 Years	4 (3.41%)	11 (3.88%)	15 (3.75%)
Marital Status	Unmarried	98 (83.79%)	215 (75.9%)	313 (78.25%)
	Married	19 (16.2%)	66 (23.3%)	85 (21.25%)
	Widow	0 (0%)	2 (0.70%)	02 (0.5%)
Education	Graduation	91(77.7%)	173 (61.1%)	264 (66%)
	Post-Graduation	20 (17.09%)	65 (22.9%)	85 (21.25%)
	Diploma	6 (5.1%)	45 (15.9%)	51 (12.75%)
Profession	Physician	102 (87.17%)	128 (45.2%)	230 (57.5%)
	Nurses	13 (11.11%)	153 (54.1%)	166 (41.5%)
	Pharmacists	1 (0.85%)	1 (0.35%)	02 (0.5%)
	Laboratory Technicians	1 (0.85%)	1 (0.35%)	02 (0.5%)
Type of ward	Tuberculosis	20 (17.1%)	35 (12.37%)	36 (9%)
	Outpatient Department	17 (14.5%)	56 (19.78%)	30 (7.5%)
	Laboratory	5 (4.27%)	8 (2.82%)	25 (6.25%)
	Medicine	18 (15.3%)	60 (21.2%)	142 (35.5%)
	Surgery	8 (6.83%)	16 (5.65%)	45 (11.25%)
	X-ray Room	4 (3.41%)	3 (1.06%)	10 (2.5%)
	Cardiology	17 (14.5%)	37 (13.07%)	27 (6.75%)
	Obstetric	11 (9.40%)	40 (14.1%)	37 (9.25%)
	Emergency	17 (14.5%)	28 (9.89%)	48 (12%)
Job Experience	Less Than 1 Year	58 (49.6%)	68 (24.02%)	126 (31.5%)
	1–2 Years	24 (20.5%)	81 (28.6%)	105 (26.25%)
	3–5 Years	24 (20.5%)	73 (25.8%)	97 (24.25%)
	6–10 Years	10 (8.5%)	33 (11.6%)	43 (10.75%)
	>10 Years	1 (0.85%)	27 (9.54%)	28 (7%)
Have you been vaccinated in last 6–12 months against influenza?	Yes	23 (19.7%)	60 (21.2%)	83 (20.75%)
	No	94 (80.3%)	223 (78.8%)	317 (79.25%)

**Table 2:** Health care professionals’ knowledge about influenza vaccination.

S. No.	Statement	Yes	No	Don’t Know
**1.**	Do you know that influenza vaccine is effective in preventing the flu?	342 (85.5%)	32 (8%)	26 (6.5%)
**2.**	Do you know that the World Health Organization (WHO) gives recommendations for influenza vaccination of Health Professionals?	303 (75.8%)	52 (13%)	45 (11.2%)
**3.**	Are you aware of the guidelines published by the WHO Advisory Committee on Immunization Practices (ACIP) for influenza immunization?	172 (43%)	169 (42.3%)	59 (14.7%)
** 4.**	Does the vaccination give 100% protection against the flu?	69 (17.3%)	242 (60.5%)	89 (22.2%)
**5.**	Does the vaccination give effective protection against upper respiratory tract infection other than influenza?	159 (39.7%)	95(23.6%)	146 (36.5%)
**6.**	Influenza vaccine can save medical costs	248 (62%)	76 (19%)	76 (19%)
**7.**	Could vaccination against influenza be a direct factor causing the flu?	136 (34%)	167 (41.7%)	97 (24.2%)
**8.**	Healthcare workers may transmit influenza to their patients	332 (83%)	40 (10%)	28 (7%)
**9.**	The influenza vaccine is composed of inactivated viruses	266 (66.5%)	37 (9.25%)	97 (24.2%)
** 10.**	The inactivated influenza vaccine does not contain live viruses that may cause some people to get influenza	236 (59%)	66 (16.5%)	97 (24.2%)
**11.**	Influenza vaccine should be administered every year	195 (48.7%)	93 (23.2%)	112 (28%)
**12.**	The appropriate time to give influenza vaccine is before flu season (December)	287 (71.7%)	36 (9%)	77 (19.2%)
**13.**	Influenza vaccines can be live or inactivated	218 (54.5%)	55(13.7%)	127 (31.7%)
**14.**	In case of mismatch of virus strains, the influenza vaccine efficacy may be reduced.	228 (57%)	70 (17.5%)	102 (25.5%)
**15.**	There is a difference between trivalent and quadrivalent influenza vaccines	225 (56.2%)	64 (16%)	111 (27.7%)

**Table 3:** Overall Knowledge, Attitude, and Practice (KAP) score: criteria for the categorization of KAP scores.

KAP Score			N (%)
**Knowledge**	High Level	≥75% (Score 34)	268 (67%)
	Low Level	<75% (Score 33)	132 (33%)
**Attitude**	Positive Attitude	Total Score > Mean (37.3)	262 (65.5%)
	Negative Attitude	Total Score < Mean (37.3)	138 (34.5%)
**Practice**	Good Practice	Total Score > Median (40)	204 (51%)
	Poor Practice	Total Score < Median (40)	196 (49%)

**Table 4:** Health care professionals’ attitude towards influenza vaccination.

S. No.	Questions	Strongly Disagree (SDA)	Disagree (DA)	Neutral (N)	Agree (A)	Strongly Agree (SA)
**1**	Do you think that vaccine is effective at preventing influenza?	23 (5.7%)	22 (5.5%)	40 (10%)	255 (63.7%)	60 (15%)
**2**	Do you think that vaccine lowers the risk of hospitalization and death?	27 (6.7%)	38 (9.5%)	65 (16.2%)	208 (52%)	62 (15.5%)
**3**	Do you think that vaccine may decrease the days of illness from influenza?	17 (4.25%)	31 (7.7%)	52 (13%)	243 (60.7%)	57 (14.2%)
**4**	Do you think that vaccinating healthcare personnel protects patients?	20 (5%)	23 (5.7%)	40 (10%)	219 (54.7%)	98 (24.5%)
**5**	Do you think that Healthcare personnel should get vaccinated for influenza every year?	34 (16%)	33 (8.25%)	43 (10.7%)	161 (40.2%)	129 (32.2%)
**6**	Do you think if vaccine provided at work place have you been vaccinated for influenza?	26 (6.5%)	39 (9.7%)	61 (15.2%)	213 (53.2%)	61 (15.2%)
**7**	Do you think if vaccine provided at home you have been vaccinated for influenza?	31 (7.75%)	31 (7.75%)	64 (16%)	201 (50.2%)	73 (18.2%)
**8**	Do you recommend the influenza vaccine to family and friends?	22 (5.5%)	27 (6.75%)	49 (12.2%)	219 (54.7%)	83 (20.7%)
**9**	Do you think that Influenza vaccine should be part of routine medical practice?	23 (6.5%)	28 (7%)	65 (16.2%)	206 (51.5%)	78 (19.5%)
**10**	Do you believe that flu vaccination of healthcare professionals will prevent influenza spread?	23 (5.75%)	29 (7.25%)	46 (11.5%)	212 (53%)	90 (22.5%)

**Table 5:** Health care professionals’ practice regarding influenza vaccination.

S. No.	Questions	Very Frequently (VF)	Frequently (F)	Occasionally (O)	Rarely (R)	Never (N)
**1**	Do you go to a health facility when you have signs of cough, colds, and sore throat?	41 (10.2%)	71 (17.7%)	118 (29.5%)	142 (35.5%)	28 (7%)
**2**	Do you use a mask when having signs of cough and cold?	89 (22.2%)	145 (36.2%)	99 (24.7%)	52 (13%)	15 (3.75%)
**3**	Do you wash your hands before and after contact with patients?	185 (46.2%)	122 (30.5%)	59 (14.7%)	30 (7.5%)	4 (1%)
**4**	Do you ever recommended to have influenza vaccination?	70 (17.5%)	104 (26%)	103 (25.7%)	61 (15.2%)	62 (15.5%)
**5**	Have you ever had influenza vaccination?	38 (9.5%)	93 (23.2%)	72 (18%)	63 (15.7%)	134 (33.5%)
**6**	Do you get influenza vaccinations on a regular basis?	16 (4%)	36 (9%)	84 (21%)	87 (21.7%)	177 (44.2%)
**7**	Have your family been vaccinated against Influenza?	40 (10%)	88 (22%)	87 (21.7%)	54 (13.5%)	131 (32.7%)
**8**	Have you ever read or attended an educational program about influenza and influenza vaccine?	14 (3.5%)	93 (23.2%)	92 (23%)	65 (16.2%)	136 (34%)

**Table 6:** Spearman’s rank correlation coefficient among knowledge, attitude, and practice.

Variables	Correlation Coefficient (rs)	*p*-Value
Knowledge and Attitude	0.19	<0.01 **
Knowledge and Practice	0.21	<0.01 **
Attitude and Practice	−0.06	0.16

** marks highly significant results.

## Data Availability

The datasets used and/or analyzed during the current study are available upon request from the corresponding author.

## Appendix A

**Table A1:** Health Care Professionals (HCPs) knowledge, attitude, and practice scores regarding influenza vaccination against gender, age, education, profession, and job experience.

Variable		High Knowledge	Low Knowledge	*p*-Value	Positive Attitude	Negative Attitude	*p*-Value	Good Practice	Poor Practice	*p*-Value
Gender	Male	63	54	<0.01 ^a^	75	42	<0.001	61	56	<0.01 ^a^
	Female	205	78		187	96		143	140	
Age	21–25 Years	152	98	0.00 ^b^	165	85	0.940 ^b^	115	135	0.008 ^b^
	26–30 Years	79	26		66	39		61	44	
	31–35 Years	25	5		19	11		18	12	
	>35 Years	12	3		12	3		10	5	
Education	Graduation	156	108	<0.01 ^b^	175	89	0.984 ^b^	116	148	0.000 ^b^
	Post-Graduation	69	16		48	37		56	29	
	Diploma	43	8		39	12		32	19	
Profession	Physician	126	104	<0.01 ^b^	149	81	0.638 ^b^	98	132	0.000 ^b^
	Nurses	139	27		109	57		104	62	
	Pharmacists	2	0		2	0		1	1	
	Laboratory Technicians	1	1		2	0		1	1	
Job Experience	Less than 1 Year	57	69	<0.01 ^b^	83	43	0.915 ^b^	60	66	0.241 ^b^
	1–2 Years	79	26		68	37		54	51	
	3–5 Years	74	23		62	35		48	49	
	6–10 Years	35	8		28	15		23	20	
	>10 Years	22	6		20	8		18	10	

Mann–Whitney U-test (Wilcox rank-sum test) ^a^ to determine the statistically significant difference between two independent groups; Kruskal–Wallis test. ^b^ to determine the statistically significant difference between three or more independent groups. *p*-value < 0.05 considered significant; <0.01 considered highly significant.

**Table A2:** Barriers to influenza vaccination.

S#	Statements	Strongly Disagree (SDA)	Disagree (DA)	Neutral (N)	Agree (A)	Strongly Agree (SA)	RII	Rank	*p* Value
**1.**	There is lack of proper storage area for vaccines that’s why Influenza vaccines are not available in the institute	36 (3.0%)	59 (14.7%)	153 (38.3%)	122 (30.5%)	30 (7.5%)	0.625	3	<0.001 *
**2.**	It is not compulsory for health care professionals to get vaccinated for Influenza	76 (19.0%)	149 (37.3%)	67 (16.7%)	87 (21.7%)	21 (5.25%)	0.514	9	0.004 *
**3.**	Influenza is not serious condition therefore not worth vaccinating against	79 (19.7%)	164 (41.0%)	69 (17.2%)	72 (18.0%)	16 (4.0%)	0.491	10	0.0001 *
**4.**	Influenza vaccine is costly that’s why not purchased normally	33 (19.7%)	76 (19.0%)	171 (42.7%)	94 (23.5%)	26 (6.5%)	0.602	5	0.889
**5.**	Not everyone is familiar with Influenza vaccination availability at their institutions	11 (2.75%)	33 (8.25%)	57 (14.3%)	222 (55.5%)	77 (19.2%)	0.760	1	0.399
**6.**	There is insufficient staff to administer vaccine	33 (8.25%)	64 (16.0%)	118 (29.5%)	142 (35.5%)	43 (10.7%)	0.649	2	<0.001 *
**7.**	Side effects and safety concerns are hindering health care professionals to get vaccinated for influenza	35 (8.75%)	83 (20.7%)	131 (32.7%)	123 (30.7%)	28 (7.0%)	0.613	4	0.0001 *
**8.**	Due to needle fear I don’t like to get vaccinated	135 (33.7%)	135 (33.7%)	46 (11.5%)	58 (14.5%)	26 (6.5%)	0.452	11	0.223
**9.**	I do not have sufficient information on the benefits of the vaccination and the consequences of the disease	39 (9.75%)	141 (35.3%)	47 (11.7%)	128 (32.0%)	45 (11.2%)	0.599	6	0.582
**10.**	I have doubts in the effectiveness of the vaccine	28 (7.0%)	133 (33.3%)	97 (24.2%)	116 (29.0%)	26 (6.5%)	0.589	7	0.003 *
**11.**	I did not have influenza vaccination for fear of the contents therein do not correspond to my belief	72 (18.0%)	141 (35.2%)	82 (20.5%)	81 (20.3%)	24 (6.0%)	0.522	8	0.008 *

*p*-value < 0.05 considered significant; * highly significant results. Fisher exact test applied between influenza vaccination in the last 6–12 months (Yes, No) and barriers to influenza vaccine (SDA, DA, N, A, SA).
